# Gut microbial biomarkers for major depressive disorder: a cross-sectional study

**DOI:** 10.3389/fcimb.2026.1690285

**Published:** 2026-07-30

**Authors:** Xuan Wang, Wei Chen, Hanlin Zhang, Di Cao, Jiaxin Sun, Huimin Hu

**Affiliations:** 1 Department of Dermatology, Lianyungang Municipal Oriental Hospital, Lianyungang, China; 2 Department of Dermatology, Jieshou City People’s Hospital, Fuyang, China; 3 Department of Dermatology, The First People’s Hospital of Lianyungang, Lianyungang, China; 4 State Key Laboratory of Pharmaceutical Biotechnology, Medical School, Nanjing University, Nanjing, Jiangsu, China; 5 Jiangsu Key Laboratory of Molecular Medicine, Medical School, Nanjing University, Nanjing, China; 6 Department of Dermatology, The Affiliated Huai’an Hospital of Xuzhou Medical University and The Second People’s Hospital of Huai’an, Huaian, China

**Keywords:** major depressive disorder, metagenome, gut microbiota, MaAsLin2, biomarkers

## Abstract

**Background:**

Alterations in the gut microbiota have been associated with a variety of psychiatric disorders, including major depressive disorder (MDD). However, the relationship between MDD and gut microbial communities remains incompletely understood. Most previous studies have primarily focused on gut bacteria, with relatively limited attention to other microbial components.

**Methods:**

In this study, we analyzed gut microbial profiles from 36 patients with MDD and 36 healthy controls using metagenomic sequencing data. The MaAsLin2 algorithm was applied to identify potential microbial biomarkers associated with MDD.

**Results:**

A total of 6 bacterial biomarkers and 7 viral biomarkers were identified. The models based on these features demonstrated strong predictive performance, with area under the curve (AUC) values of 0.891 for bacteria and 0.878 for viruses. Notably, the combined bacterial-viral model achieved an AUC of 0.946. These findings were further evaluated through external testing in two unrelated research cohorts. In the Shanxi cohort, the AUC values were 0.825 (bacteria), 0.803 (viruses), and 0.972 (combined model). In the Wuhan cohort, the AUC values were 0.683 (bacteria), 0.693 (viruses), and 0.784 (combined model).

**Conclusion:**

In summary, our results highlight the potential of gut bacterial and viral biomarkers as candidate biomarkers and potential auxiliary tools for MDD assessment and suggest that integrating multi-domain microbial features may improve prediction accuracy.

## Introduction

Major depressive disorder (MDD) is a global psychiatric disorder with symptoms that include persistent low mood, lack of pleasure or diminished interest, poor concentration, changes in appetite, sleep disturbances, and even suicide. The prevalence of MDD is 3.8%, and the number of people with the disorder now exceeds 280 million globally. MDD is characterized by a high rate of recurrence and a high rate of suicidality, with about 10% to 15% of patients committing suicide and about two-thirds of patients with MDD consider suicide. The pathogenesis of MDD is unclear and specific biomarkers are lacking (Marx et al., 2023; Jiao et al., 2025).

MDD is a highly heterogeneous disease with considerable inter-individual variation in clinical symptoms and disease severity. Diagnosing MDD faces multifaceted challenges, primarily because of the subjective nature of current diagnostic methods. Clinically, psychiatric disorders are assessed primarily through patient history and mental status examinations, as well as standardized self-assessment scales (Paykel, 2008). This diagnostic approach is susceptible to subjective bias. Heterogeneity and subjectivity complicate the diagnosis of MDD and increase the rate of misdiagnosis, with about a quarter of patients with bipolar disorder misdiagnosed with MDD (Yin and Li, 2025). Therefore, the search for objective biomarkers of MDD is a very promising study.

A growing body of research has focused on the association between gut microbiota and MDD. Previous studies have mainly characterized bacterial alterations, such as changes in Firmicutes, Actinobacteria, and Bacteroidetes, as well as genus-level shifts including increased Bacteroides and decreased Blautia, Faecalibacterium and Coprococcus (Liu et al., 2023). In addition, certain taxa such as Alistipes and Oscillibacter have been linked to inflammatory processes and depressive symptoms (Naseribafrouei et al., 2014). In terms of viruses, the abundances of Clostridium_phage_phi8074-B1, Klebsiella_phage_vB_KpnP_SU552A, and Escherichia_phage_ECBP5 increased in MDD (Yang et al., 2020). However, the relationship between MDD and the gut microbiota has not yet fully clarified. At the same time, related research faces many challenges, such as the reliability of results from different cohorts, and the research on viruses in the gut of MDD patients is still insufficient. In this study, we systematically analyzed gut microbial communities, including bacteria, viruses, and fungi, in patients with MDD and healthy controls. Based on these data, we identified microbial features associated with MDD and constructed prediction model using the MaAsLin2 algorithm. Furthermore, we evaluated the predictive performance of these microbial biomarkers through external testing in two unrelated research cohorts from different geographic regions.

## Methods

### Data collection

In this study, the unassembled raw metagenomic sequencing data were available from the NCBI (SRA accession numbers: PRJNA762199, PRJNA1083304 and PRJNA943232). The Russian training cohort (PRJNA762199) included 36 patients with MDD 36 healthy individuals. Case - control matching was carried out on the basis of age, gender, and body mass index (BMI). The Shanxi validation cohort (PRJNA1083304) included 16 patients with MDD and 20 healthy individuals. The Wuhan validation cohort (PRJNA943232) included 40 patients with MDD and 42 healthy individuals. Diagnosis of MDD was derived using the International Classification of Diseases - 10th revision (ICD-10). All patients met the diagnostic criteria for MDD, and the severity of depression was assessed using the HAMD-17. And all of these patients were first onset MDD who had not received medication and had no history of substance abuse. The control group consisted of healthy volunteers, whose inclusion criteria were: no history of mental illness, no history of severe traumatic brain injury, no diseases related to the use of psychoactive substances (alcohol, drugs). All subjects who took antibiotics or probiotics within 3 months prior to fecal collection were excluded.

### Data processing

Raw data in SRA format were converted into FASTQ format using the fastq-dump command of the SRA toolkit. FASTQC was used to assess the quality of sequenced reads. Low quality reads, adapter contamination, and human DNA contamination were removed using Kneaddata software to obtain clean reads. Clean reads were then classified using Kraken2 based on bacterial, viral and fungal databases. For the kmer-based identification, a standard Kraken2 database was used. Finally Bracken was used to estimate the relative abundance of bacteria, viruses and fungi in each sample.

### Data visualization

Microbial abundance were adjusted using MMUPHin, a zero-inflated empirical Bayes framework and normalized using the phyloseq package while filtering out the genera with relative abundance below 0.01%. Stacked bar charts were plotted in R (version 4.3.1) using the ggtree and ggplot2 packages. Differences in microbial communities between the MDD groups and the healthy controls were analyzed using MaAsLin2. Collinearity among the independent variables was tested by the collinearity statistics in the SPSS software, including tolerance and variance expansion factor (VIF). Statistical analysis was performed using SPSS (version 21.0) and ROC curves were drawn using MedCalc (version 15.2.2).

## Results

### Clinical characteristics of patients with major depressive disorder

To investigate the association between MDD and gut microbiota, we included multiple metegenomic sequencing data from 190 individuals from different regions and countries, including 98 healthy controls and 92 patients with MDD. In the training cohort (including 36 MDDs and 36 healthy individuals), no significant differences were found in age (30.83 vs 33.97 on average), gender and body mass index (BMI) (22.39 vs 24.63 on average) between MDDs and healthy controls (p > 0.05). The MDD groups had significantly higher Hamilton Depression Rating Scale (HAMD-17) scores than the HC groups (21.33 vs 1.64 on average; P < 0.01). In Shanxi validation cohort (including 16 MDDs and 20 healthy individuals), the difference between MDDs and healthy controls in terms of gender and body mass index (BMI) (21.68 vs 22.17 on average) was not statistically significant (P > 0.05). The difference in age between MDDs and healthy controls was statistically significant (20.19 vs 23.50 on average; P < 0.05), but the difference in mean age between the two groups was only 3.31 years. We assessed patients’ depression levels using the Hamilton Depression Rating Scale (HAMD-17), and the results were statistically significant (26.31 vs 1.85 on average; P < 0.001). In the Wuhan validation cohort, there were no significant differences between MDDs and healthy controls in terms of age (21.15 vs 22.00 on average), gender and BMI (20.02 vs 20.71 on average; P > 0.05) (**Table 1**).

**Table 1:** Clinical characteristics in MDD and HCs.

Group	Variable	Sex(male/female)	Age(years)	BMI	HAMD-17
Training cohort(Russian)	MDD (n = 36)	19/17	30.83 ± 10.769	22.39 ± 4.680	21.33 ± 3.610
	HCs (n = 36)	19/17	33.97 ± 10.476	24.63 ± 4.765	1.64 ± 1.944
	t/χ2	0	1.254	1.866	28.822
	P	1.000^a^	0.214^b^	0.067^b^	0.000^b^
Testing cohort(Shanxi)	MDD (n = 16)	8/8	20.19 ± 4.230	21.68 ± 3.626	26.31 ± 6.620
	HCs (n = 20)	7/13	23.50 ± 3.348	22.17 ± 2.115	1.85 ± 2.720
	t/χ2	–	2.625	0.507	15.054
	P	0.500^a^	0.013^b^	0.615^b^	0.000^b^
Testing cohort(Wuhan)	MDD (n = 40)	5/35	21.15 ± 2.723	20.02 ± 2.114	–
	HCs (n = 42)	8/34	22.00 ± 2.828	20.71 ± 2.253	–
	t/χ2	0.658	1.385	1.42	–
	P	0.417^a^	0.170^b^	0.160^b^	–

^a^ P value for chi-square test.

^b^ P values for two-sample t-test.

### Gut microbial markers in patients with major depressive disorder

**Figure 1:**
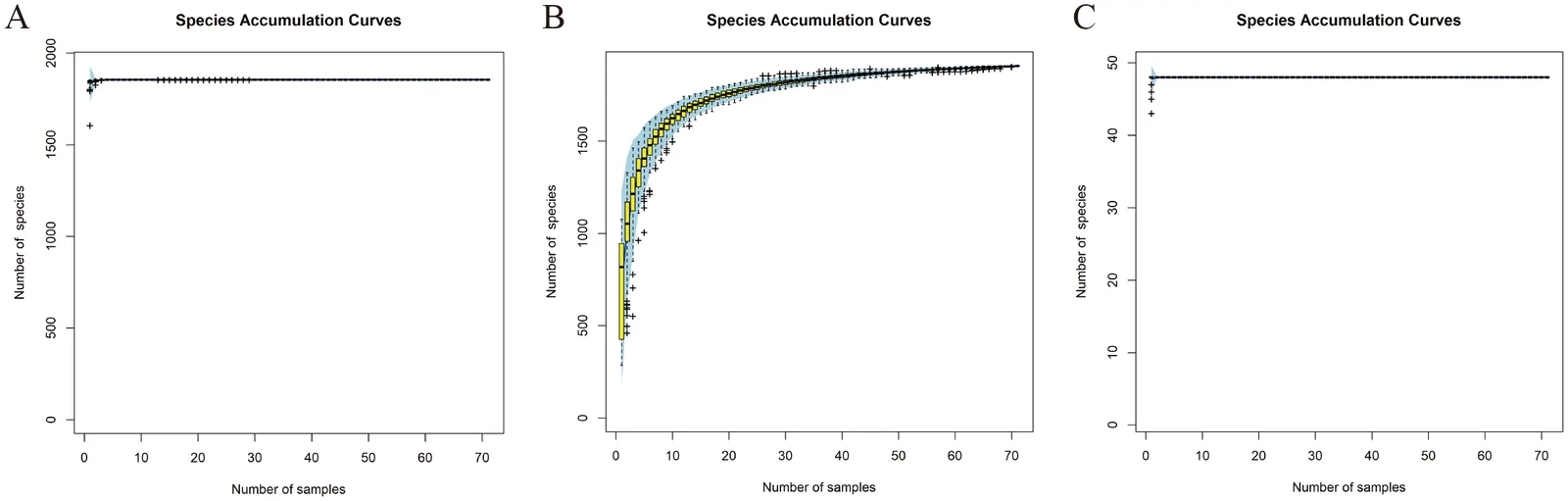
**(A)** Species accumulation curve of gut bacteria. **(B)** Species accumulation curve of gut virus. **(C)** Species accumulation curve of gut fungi.

Firstly, we conducted batch correction, and MMUPHin successfully reduced the batch effects among different studies (**Supplementary Figures 1**–**3**). The species accumulation curve is used to determine whether the sample size is sufficient for high-throughput community analysis of species. If the trend of this curve is steep, it indicates that the sample size is too small. As the sample size increases, the upward trend of the curve becomes relatively gentle, indicating that the sample size is large enough for subsequent analysis. The results show that the curve in this study has reached a plateau, indicating that the sample size is sufficient to reflect the composition and diversity of the intestinal microbiota (**Figures 1A–C**).

**Figure 2:**
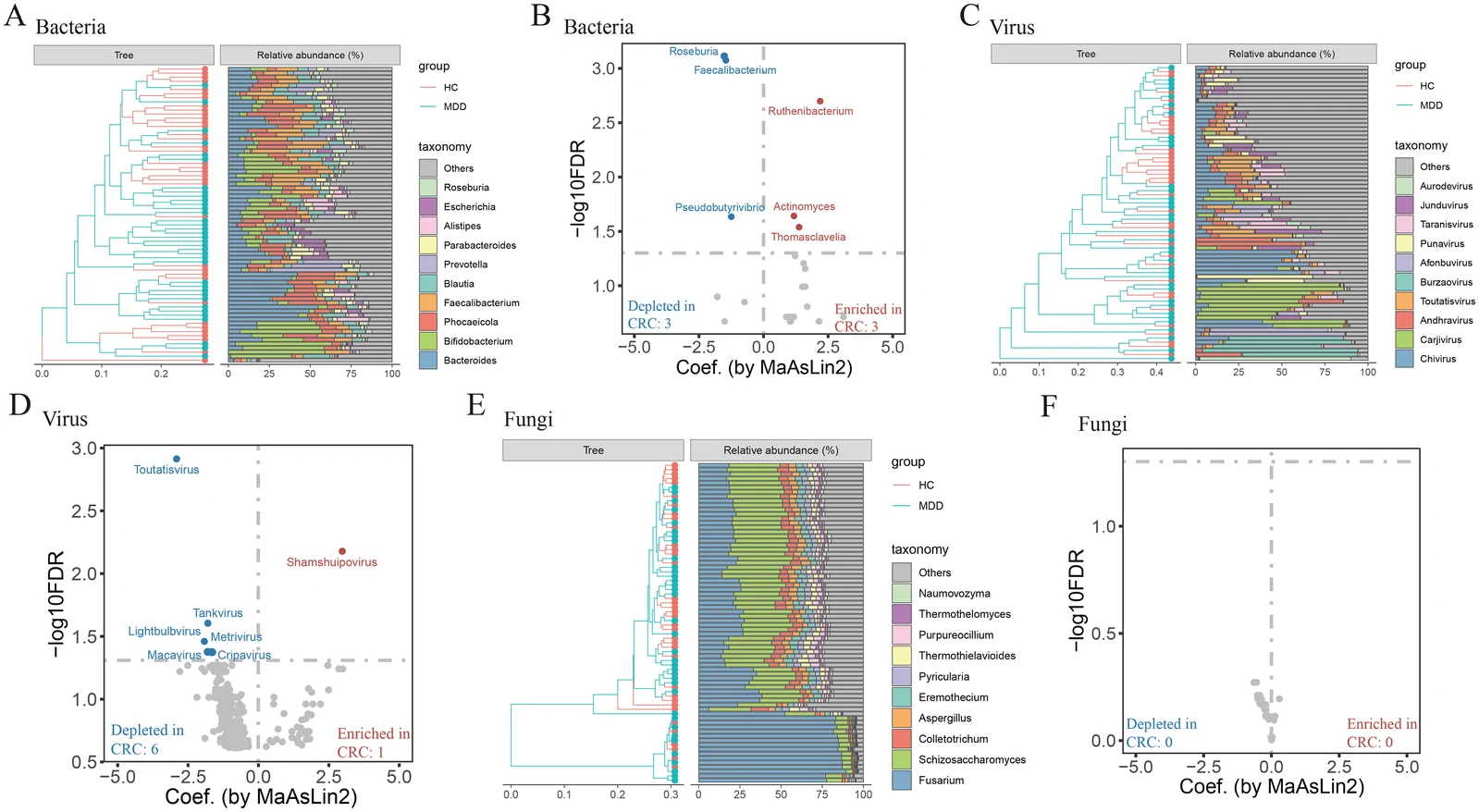
**(A)** Stacked bar plot of gut bacteria composition at the genus level. **(B)** Volcano plots show the associations between gut bacteria and MDD calculated using MaAsLin2. **(C)** Stacked bar plot of gut viruses composition at the genus level. **(D)** Volcano plots show the associations between gut viruses and MDD calculated using MaAsLin2. **(E)** Stacked bar plot of gut fungi composition at the genus level. **(F)** Volcano plots show the associations between gut fungi and MDD calculated using MaAsLin2.

The stacked bar graph shows the relative abundance of species and the stacked area plot indicates the species composition in each individual, showing the top ten species in terms of relative abundance. To further explore the changes in the microbial components and the correlation between the abundance of gut microbes and MDD, multivariate regression analysis was performed using the MaAsLin2 R package. A total of 6 gut bacterial biomarkers and 7 gut viral biomarkers were identified. However, we did not identify gut fungal biomarkers associated with the prediction of MDD. Four microbial communities were significantly enriched in MDD groups compared to healthy controls, including Ruthenibacterium, Actinomyces, Thomasclavelia, and Shamshuipovirus. Whereas, there were 9 microbial communities significantly decreased, including Roseburia, Faecalibacterium, Pseudobutyrivibrio, Toutatisvirus, Tankvirus, Lightbulbvirus, Cripavirus, Macavirus, and Metrivirus (**Figures 2A–F**). We tested the variables for multicollinearity by calculating tolerance and variance inflation factor (VIF). The results showed that the tolerance was > 0.1 and VIF <10, indicating that there was no significant multicollinearity between all independent variables (**Table 2**).

**Table 2:** Tolerance and variance inflation factor.

Variables	Tolerance	VIF
Roseburia	0.451	2.217
Faecalibacterium	0.457	2.19
Ruthenibacterium	0.797	1.255
Actinomyces	0.722	1.384
Pseudobutyrivibrio	0.474	2.109
Thomasclavelia	0.87	1.149
Toutatisvirus	0.493	2.027
Shamshuipovirus	0.767	1.303
Tankvirus	0.464	2.156
Lightbulbvirus	0.31	3.226
Cripavirus	0.651	1.537
Macavirus	0.529	1.889
Metrivirus	0.529	1.891

**Figure 3:**
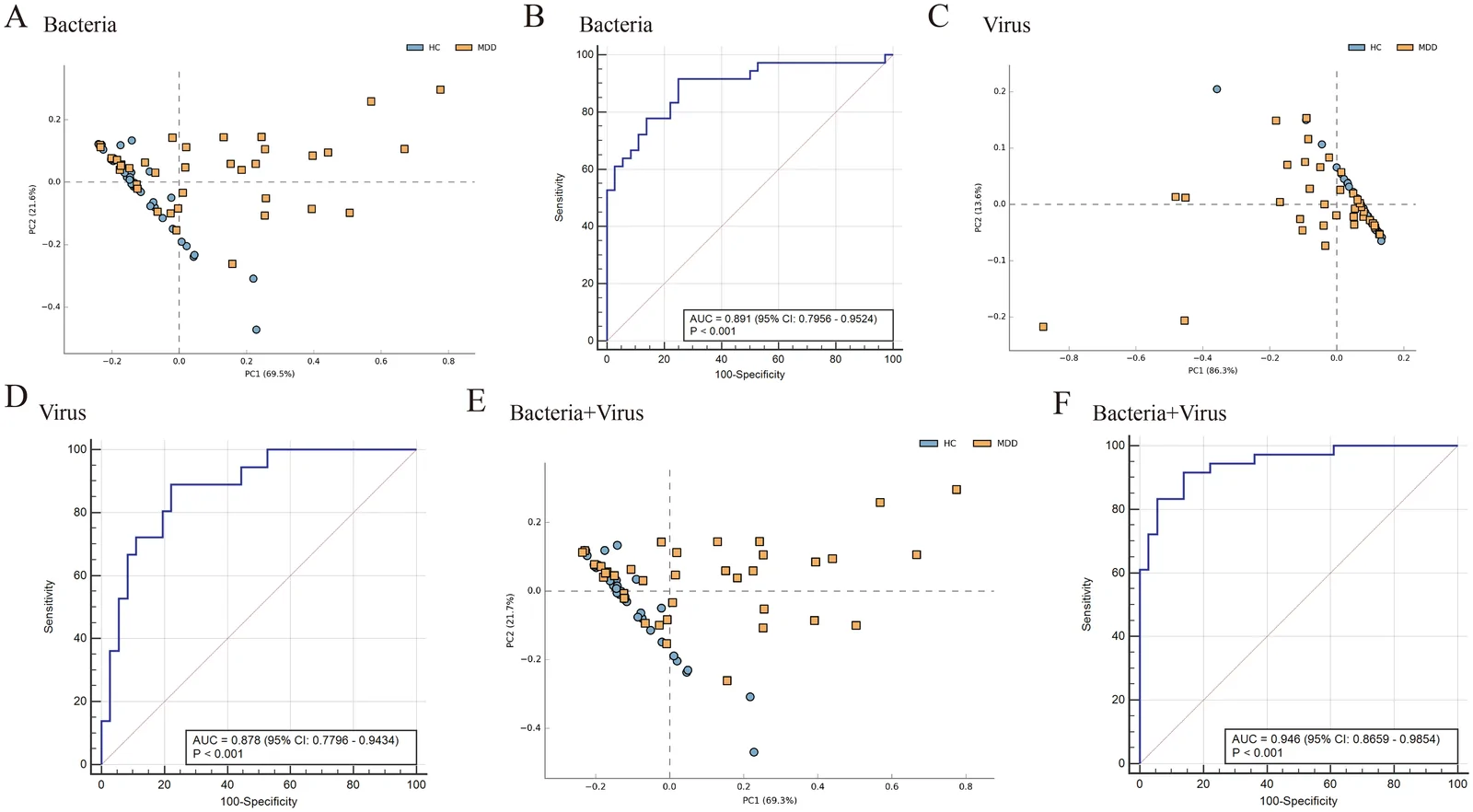
**(A)** PCoA of the characteristic bacterial communities. **(B)** ROC curve of gut bacteria in training cohort. **(C)** PCoA of the characteristic viral communities. **(D)** ROC curve of gut viruses in training cohort. **(E)** PCoA of the characteristic combined bacterial-viral communities. **(F)** ROC curve of gut multi-kingdom microbiota (bacteria and viruses) in training cohort.

Finally, we assessed the predictive performance of gut microbiota for MDD by receiver operating characteristic (ROC) curves. In the training cohort, we first tested the model accuracy of using single-kingdom microbial biomarkers to distinguish between MDD and healthy individuals. The results showed good predictive performance of the models constructed by the MaAsLin2 algorithm, with the areas under the ROC curves (AUC) values as high as 0.891 (95% CI: 0.7956 - 0.9524) for the model based on bacteria and the AUC value of 0.878 (95% CI: 0.7796 - 0.9434) for the model based on virus, suggesting that these characteristic microbial communities can be used as the biomarkers for MDD (**Figures 3A–D**). We next explored the performance of the combined multi-kingdom microbial prediction model. We found that the multi-kingdom microbial model performed superiorly in MDD with an AUC of 0.946 (95% CI: 0.8659 - 0.9854), which was superior compared to the single-kingdom microbial model (**Figures 3E, F**). These results confirm that multi-kingdom gut microbial markers have higher predictive performance in MDD than single kingdom.

**Figure 4:**
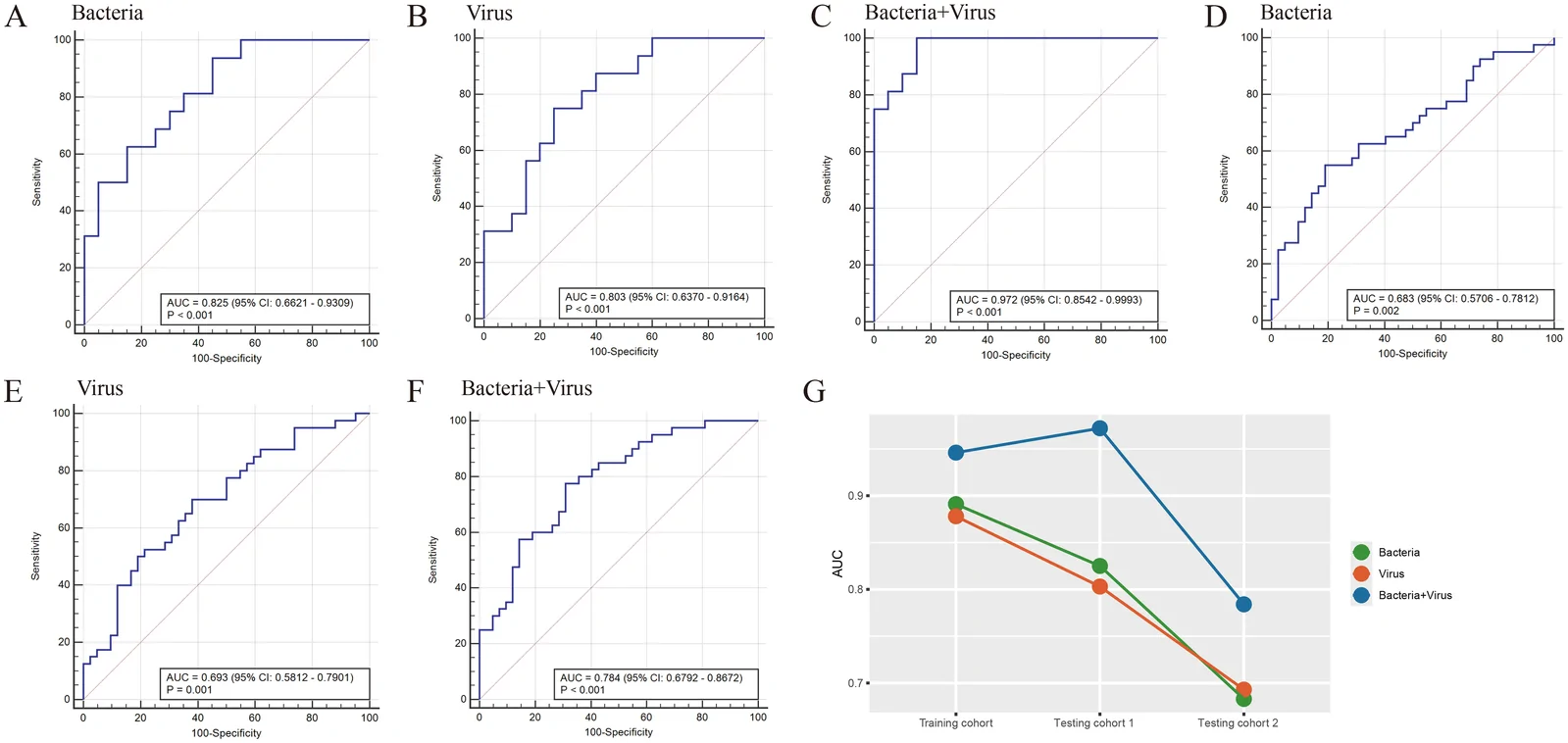
**(A)** ROC curve of gut bacteria in Testing cohort from Shanxi. **(B)** ROC curve of gut viruses in Testing cohort from Shanxi. **(C)** ROC curve of gut multi-kingdom microbiota (bacteria and viruses) in Testing cohort from Shanxi. **(D)** ROC curve of gut bacteria in Testing cohort from Wuhan. **(E)** ROC curve of gut viruses in Testing cohort from Wuhan. **(F)** ROC curve of gut multi-kingdom microbiota (bacteria and viruses) in Testing cohort from Wuhan. **(G)** Comparison of ROC curves in different groups.

To further evaluate the predictive performance of microbial communities, we conducted external testing in two unrelated research cohorts from Shanxi and Wuhan to assess the reliability of the model. In the Shanxi cohort, the AUC of the model based on bacteria was 0.825 (95% CI: 0.6621 - 0.9309), the AUC of the model based on virus was 0.803 (95% CI: 0.6370 - 0.9164), and the AUC of the multi-kingdom microbial model was 0.972 (95% CI: 0.8542 - 0.9993) (**Figures 4A–C**). In the Wuhan cohort, the AUC of the model based on bacteria was 0.683 (95% CI: 0.5706 - 0.7812), the AUC of the model based on virus was 0.693 (95% CI: 0.5812 - 0.7901), and the AUC of the multi-kingdom microbial model was 0.784 (95% CI: 0.6792 - 0.8672) (**Table 3**; **Figures 4D–F**). Therefore, we can conclude that the gut microbiota can serve as a biomarker for MDD, and the predictive performance of the multi-kingdom microbial model was significantly better than that of the single-kingdom microbial model (**Figure 4G**).

**Table 3:** The predictive performance of the microbial model.

Group	Model	AUC (95% CI)	p-value
Training cohort(Russian)	Bacteria	0.891 (95% CI: 0.7956 - 0.9524)	P < 0.001
	Virus	0.878 (95% Cl: 0.7796 - 0.9434)	P < 0.001
	Bacteria+Virus	0.946 (95% Cl: 0.8659 - 0.9854)	P < 0.001
Testing cohort(Shanxi)	Bacteria	0.825 (95% CI: 0.6621 - 0.9309)	P < 0.001
	Virus	0.803 (95% CI: 0.6370 - 0.9164)	P < 0.001
	Bacteria+Virus	0.972 (95% CI: 0.8542 - 0.9993)	P < 0.001
Testing cohort(Wuhan)	Bacteria	0.683 (95% CI: 0.5706 - 0.7812)	P = 0.002
	Virus	0.693 (95% CI: 0.5812 - 0.7901)	P = 0.001
	Bacteria+Virus	0.784 (95% CI: 0.6792 - 0.8672)	P < 0.001

## Discussion

Given the inherent subjectivity and attendant risk of misdiagnosis in MDD, the identification of objective biomarkers is imperative. Using the MaAsLin2 algorithm, we established a predictive model for MDD and identified 6 bacterial and 7 viral biomarkers.

Among the 6 bacterial biomarkers, significant enrichment was observed for Ruthenibacterium, Actinomyces, and Thomasclavelia, whereas Roseburia, Faecalibacterium, Pseudobutyrivibrio were significantly reduced. Accumulating evidence has identified bacteria of the genus Ruthenibacterium as potential pathogens in COVID-19 patients, where their presence correlates with clinical manifestations including reduced circulating immune cell counts and refractory hypoxemia (Yu et al., 2020). Parallel gut microbiota analyses in people with depression have further revealed significant enrichment of Ruthenibacterium lactatiformans and Escherichia coli in this patient cohort (Kovtun et al., 2022). High-throughput pyrosequencing studies have additionally demonstrated that the relative abundance of Actinomycetes is markedly elevated in the fecal microbial communities of individuals with MDD (Naseribafrouei et al., 2014; Jiang et al., 2015; Chen et al., 2018; Huang et al., 2019). In children with autism spectrum disorder (ASD), metagenomic profiling shows an increased Firmicutes/Bacteroidetes ratio compared with typically developing (TD) peers, accompanied by a particularly prominent expansion of the genus Thomasclavelia (Valencia-Buitrago et al., 2025). In contrast, consistent depletion of two core human gut short-chain fatty acid (SCFA)-producing taxa — Faecalibacterium and Roseburia — has emerged as a shared microbial signature across multiple neuropsychiatric conditions, including depression, anxiety, schizophrenia and autism (Hawkins et al., 2025). This reduction is robustly validated in metagenomic datasets from people with depression, and is mechanistically linked to disease pathogenesis through two complementary pathways. First, MDD is well-established to be associated with a state of low-grade chronic systemic inflammation, a context in which the documented potent anti-inflammatory properties of Faecalibacterium are likely to be functionally relevant (Sokol et al., 2008). Second, the loss of these butyrate-producing bacteria directly diminishes intestinal butyrate biosynthesis, compromising gut barrier integrity. This impairment facilitates translocation of lipopolysaccharide (LPS) into the systemic circulation, where it activates the TLR4/NF-κB signaling cascade to drive elevated production of pro-inflammatory cytokines such as IL-6 and TNF-α (Šimunović Filipčić et al., 2025; Hawkins et al., 2025; Cao et al., 2026). The downstream consequences of this cascade include the induction of neuroinflammation and dysregulation of the hypothalamic–pituitary–adrenal (HPA) axis stress response, both of which exacerbate depressive and anxiety-related behavioral phenotypes. A gut microbiota study of idiopathic inflammatory myopathies has documented a significant depletion of several butyrate-producing bacteria, including members of the Pseudobutyrivibrio family, in patient cohorts. Following IL-2 immunotherapy, these taxa exhibit a marked rebound in abundance. This compositional shift shows a strong positive correlation with concurrent elevations in serum L-aspartate and L-leucine levels (Zhufeng et al., 2022). These findings collectively reinforce the hypothesis that restoration of butyrate-producing bacterial populations is tightly coupled with the resolution of inflammatory status.

Investigations of the human enteric virome remain in their infancy. Although viral communities constitute a central component of the gut microecosystem, they have historically been under-investigated. Current evidence indicates that the gut virome is dominated by bacteriophages, the overwhelming majority of which are temperate; their estimated collective load reaches 1×10^15^ particles and their replication is strictly dependent on bacterial hosts (Shkoporov and Hill, 2019; Gregory et al., 2020; Wang et al., 2022). Bacteriophage-bacterium interactions, together with direct viral effects on the host, have been causally linked to the pathogenesis of multiple diseases, positioning the enteric virome as a promising biomarkers and therapeutic targets (Nakatsu et al., 2018; Tetz and Tetz, 2018; Duan et al., 2019). In the present study, patients with MDD exhibited distinct viral signatures: Shamshuipovirus was significantly enriched, whereas Toutatisvirus, Tankvirus, Lightbulbvirus, Cripavirus, Macavirus, and Metrivirus were markedly depleted.

**Figure 5:**
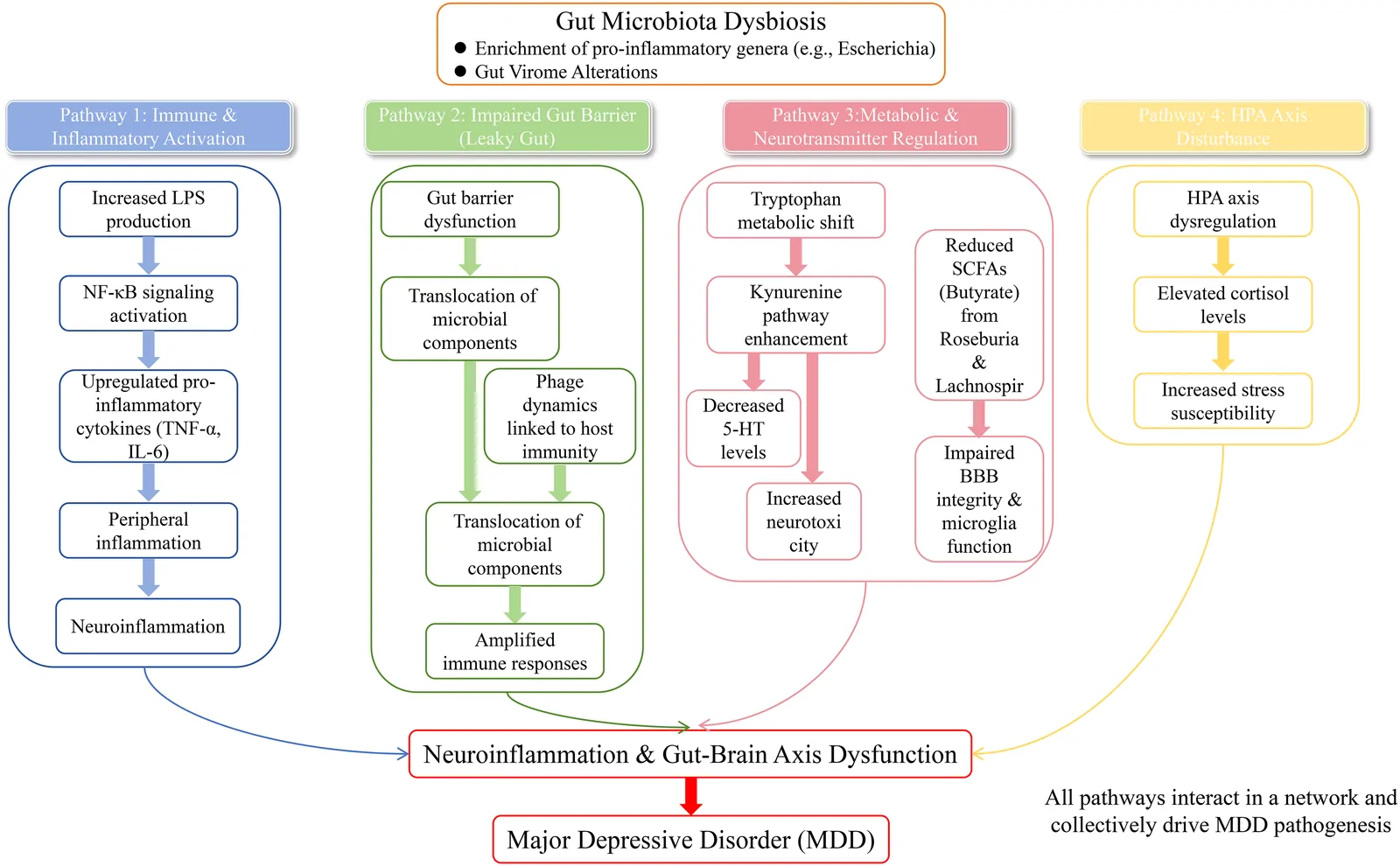
Association between major depressive disorder and gut microbiota dysbiosis.

Based on the above findings, we propose a hypothesis that the gut microbiota may contribute to the pathogenesis and progression of MDD through multiple interconnected pathways. However, it should be noted that the findings of this study reflect associations, and it remains unclear whether these microbial alterations contribute to the onset of MDD or occur as a consequence of the disorder. First, microbial dysbiosis can trigger lipopolysaccharide (LPS)-mediated immune-inflammatory responses, activating the NF-κB pathway and upregulating pro-inflammatory cytokines such as TNF-α and IL-6, thereby promoting neuroinflammation (Song et al., 2025). Second, impaired intestinal barrier function not only facilitates the translocation of bacterial components into systemic circulation but may also enable the translocation of bacteriophages or their nucleic acids across the barrier (Bonaz and Bernstein, 2013; Capuco et al., 2020; Khan et al., 2026). This process could further amplify immune responses via receptors such as TLR3 and TLR7, thereby integrating alterations of the gut virome into host immune regulatory networks (Norman et al., 2015). In addition, dysbiosis may disrupt tryptophan metabolism by shifting it from serotonin (5-HT) synthesis toward the kynurenine pathway, while simultaneously reducing the production of short-chain fatty acids (SCFAs) by genera such as Roseburia and Lachnospira, ultimately affecting neuroinflammation and blood-brain barrier function (Zhang et al., 2025). Meanwhile, the gut microbiota may also enhance host stress responsiveness through modulation of the hypothalamic-pituitary-adrenal (HPA) axis (Bertollo et al., 2025). Notably, recent studies have indicated that environmental factors, such as hypobaric hypoxia at high altitudes, may modulate depression-related pathways by altering the gut microbiota, suggesting a potential regulatory role of the gut–brain axis mediated by the interaction between environmental conditions and microbial communities (**Figure 5**) (Qiao et al., 2025).

For the first time, we integrated bacterial and viral biomarkers into a dual-domain predictive model for MDD. Although the cohort has been matched for age, gender, and BMI, residual confounding effects may still exist due to factors such as smoking, alcohol consumption, dietary habits, sleep patterns, exercise habits, and educational level. Future studies should comprehensively collect information on these variables to minimize residual confounding as much as possible. Given the limited sample size, particularly in the context of multi-kingdom analyses, statistical power may be constrained and effect sizes attenuated; therefore, the findings should be considered exploratory. Nonetheless, they provide novel, evidence-based insights into the gut microbiota-MDD axis and establish both methodological and theoretical foundations for future large-scale, multi-omic longitudinal investigations.

## Data Availability

The datasets presented in this study can be found in online repositories. The names of the repository/repositories and accession number(s) can be found below: https://www.ncbi.nlm.nih.gov/, PRJNA762199 https://www.ncbi.nlm.nih.gov/, PRJNA1083304 https://www.ncbi.nlm.nih.gov/, PRJNA943232.

## Supplementary material

Supplementary Figure 1 Bacterial batch correction. PCoA before **(A, C)** and after **(B, D)** MMUPHin correction.

Supplementary Figure 2 Viral batch correction. PCoA before **(A, C)** and after **(B, D)** MMUPHin correction.

Supplementary Figure 3 Fungal batch correction. PCoA before **(A, C)** and after **(B, D)** MMUPHin correction.
