# Correction: Gut microbial biomarkers for major depressive disorder: a cross-sectional study

**DOI:** 10.3389/fcimb.2026.1978800

**Published:** 2026-09-09

**Authors:** Xuan Wang, Wei Chen, Hanlin Zhang, Di Cao, Jiaxin Sun, Huimin Hu

**Affiliations:** 1 Department of Dermatology, Lianyungang Municipal Oriental Hospital, Lianyungang, China; 2 Department of Dermatology, Jieshou City People’s Hospital, Fuyang, China; 3 Department of Dermatology, The First People’s Hospital of Lianyungang, Lianyungang, China; 4 State Key Laboratory of Pharmaceutical Biotechnology, Medical School, Nanjing University, Nanjing, Jiangsu, China; 5 Jiangsu Key Laboratory of Molecular Medicine, Medical School, Nanjing University, Nanjing, China; 6 Department of Dermatology, The Affiliated Huai’an Hospital of Xuzhou Medical University and The Second People’s Hospital of Huai’an, Huaian, China

**Keywords:** major depressive disorder, metagenome, gut microbiota, MaAsLin2, biomarkers

There was a mistake in **Figure 2** as published. There are errors in the disease abbreviations in **Figures 2B, D, F**. The corrected **Figure 2** appears below.

**Figure 2:**
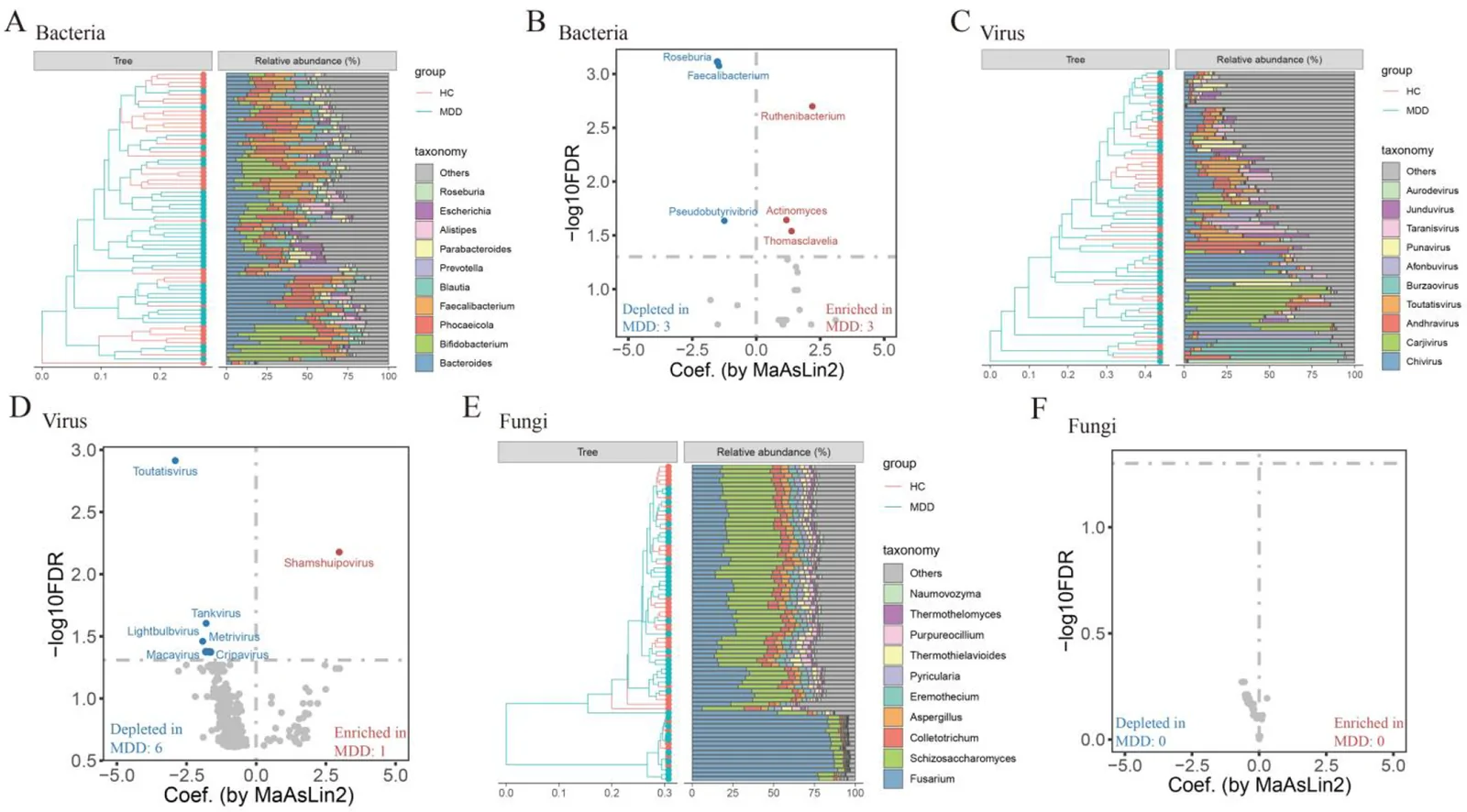
**(A)** Stacked bar plot of gut bacteria composition at the genus level. **(B)** Volcano plots show the associations between gut bacteria and MDD calculated using MaAsLin2. **(C)** Stacked bar plot of gut viruses composition at the genus level. **(D)** Volcano plots show the associations between gut viruses and MDD calculated using MaAsLin2. **(E)** Stacked bar plot of gut fungi composition at the genus level. **(F)** Volcano plots show the associations between gut fungi and MDD calculated using MaAsLin2.

The original version of this article has been updated.

